# Acuities into tolerance mechanisms via different bioassay during *Brassicaceae-Alternaria brassicicola* interaction and its impact on yield

**DOI:** 10.1371/journal.pone.0242545

**Published:** 2020-12-01

**Authors:** Sana Munir, Ahmad Naeem Shahzad, Muhammad Kamran Qureshi

**Affiliations:** 1 Department of Plant Breeding and Genetics, Faculty of Agricultural Sciences and Technology, Bahauddin Zakariya University, Multan, Pakistan; 2 Department of Agronomy, Faculty of Agricultural Sciences and Technology, Bahauddin Zakariya University, Multan, Pakistan; Harran University, TURKEY

## Abstract

Heavy losses by dark leaf spot disease in oilseed *Brassica* have incited research towards identifying sources of genetic tolerance against causal pathogen, *Alternaria brassicicola*. Several morpho-molecular parameters were evaluated to test the performance of field mustard and rapeseed genotypes under artificial inoculation with this pathogen. During *Brassica*-*Alternaria* interaction, physio-biochemical defense response was witnessed in tolerant genotypes. Two tolerant genotypes (one for field mustard and one for rapeseed), i.e., EC250407 and EC1494 were identified. However, necrotic lesions were more prominent in susceptible genotypes with minimum chlorophyll (chlorophyll *a*, chlorophyll *b* and total chlorophyll) and carotenoids contents. Contrary to photosynthetic pigments, increase in total soluble protein (TSP) contents was observed with disease progression in susceptible genotypes. Tolerant genotypes of field mustard and rapeseed displayed remarkable increase in the activities of redox enzyme in infected leaves with least yield loss (6.47% and 5.74%) and disease severity index (DSI) of 2.9 and 2.1, respectively. However, yield/plant showed close association with other morpho-yield parameters, photosynthetic pigments and redox enzymes (superoxide dismutase (SOD), catalase (CAT) and peroxidase (POD)) activities except silique length and TSP. Based on the results of morpho-biochemical analyses, redox enzymes and morphological parameters; their interplay is proposed to determine the tolerance outcome of the *Brassica*-*A*. *brassicicola* interaction.

## Introduction

*Brassica* (*Brassica napus*, *B*. *juncea* and *B*. *rapa*) ranks second among oilseed crops after soybean [1]. The genus *Brassica* consists of members rich in nutritional and economical values [2]. In spite of substantial crop production in oilseed *Brassica*, immense inconsistency remains between actual and potential yield due to exposure to various stresses (biotic/abiotic) [3]. *Alternaria* fungi are well-known among biotic stresses for their damaging behavior [4]. These fungi are the cause of numerous diseases in ~400 plant species [5], mainly members of *Brassica* family like *B*. *oleraceae* (Cabbage), *B*. *nigra* (Black mustard) and *B*. *rapa* (Mustard) [4]. Dark leaf spot triggered by *A*. *brassicicola* causes 23–57% yield reduction in *Brassicaceae* family [6], by infecting seedlings, seeds and other edible parts [7,8]. Thus, creating a challenge in global production of edible *Brassica*, including both vegetable and oilseed [7].

The host plant infected with *A*. *brassicicola* develop typical symptoms during developmental stages such as dark brown lesions with concentric rings surrounded by yellow halo on leaves, stem and siliques. Under optimum conditions, these necrotic lesions cause severe reduction in photosynthetic efficiency, accelerate plant senescence and lead to plant death causing crop losses [9,10]. Additionally, successful infection also leads to disruption of cell wall proteins and overproduction of reactive oxygen species (ROS) [11]. The ROS play an integral role in various mechanisms ranging from developmental to defense processes in plants and are often linked with disease tolerance [12]. However, overproduction of ROS affects developmental and physiological processes by damaging cell membrane, proteins and photosynthetic machinery, i.e., carotenoids and chloroplast in host plant [13]. Antioxidant guard mechanism is the most protuberant response towards these increased ROS molecules by acting as scavengers. Failure of these antioxidants in ROS scavenging results in “oxidative stress” [14].

Plant susceptibility to necrotrophic fungi such as *A*. *brassicicola* relies on balance between ROS generation and its scavenging via antioxidant defense mechanism [15]. Imbalance between ROS and its scavenging machinery reflect failure of host defense strategy or successful pathogen infection. The induction of ROS detoxifying enzymes, such as CAT, SOD and POD are the most common ROS scavenging mechanism during stress response [16] contributing towards plant tolerance.

To augment pathogen control, a tolerant cultigen enriched with defense capacity for *Brassicaceae* crops is required. Unfortunately, to date there is no significant contributions for tolerance in *Brassica* (*Alternaria*-tolerant variety) exist. Additionally, tolerant wild *Brassica* plants do not outbreed well with the domesticated susceptible ones [9,10].

Successful breeding program relies on methods used to distinguish genetic variations in tolerance at early plant developmental stages. To develop pathogen tolerant varieties, it is inevitable to find sources of *Alternaria* tolerance in *Brassica* germplasm. The key goal of current study was to find such sources in rapeseed and field mustard germplasm. The study was designed to evaluate the role of bioassays regarding photosynthetic pigments, total soluble proteins as well as redox enzymatic activities at different time intervals, i.e., hours post infection (hpi) in rapeseed and field mustard towards disease tolerance. Subsequent biochemical analysis with morphological markers and association between them during the course of infection aided to conclude the tolerance mechanisms in *Brassica* plants.

## Materials and methods

### Plant and pathogen material

*Brassicaceae* germplasm used in the current study included genotypes of *B*. *rapa* and *B*. *napus* collected from National Agricultural Research Centre, Islamabad, Pakistan (Table 1). Genotypes were sown under open field/natural conditions at research farm, Department of Plant Breeding and Genetics, Faculty of Agricultural Sciences and Technology (FAS&T), Bahauddin Zakariya University, Multan, Pakistan, under recommended cultural practices.

**Table 1:** *Brassica* genotypes used in the current study, their names, accession numbers, DSI ± SD, vegetation and maturity period, species and genetic background/origin (if known).

Genotypes^a^	DSI ± SD^b^	Veg. Period (Days)^c^	Maturity Period (Days)^d^	Genetic Background/Origin^e^
**EC25047**	2.9 ± 0.19	40	105	*Brassica rapa* subsp. *oleifera* (USA)
**EC1333**	9.5 ± 0.39	40	105	*Brassica rapa* subsp. *oleifera* (Pakistan)
**EC1494**	2.1 ± 0.19	50	125	*Brassica napus* subsp. *napus* (Pakistan)
**EC24181**	5 ± 0.33	50	125	*Brassica napus* subsp. *napus*

^a^ genotype’s commercial names or accession number used during study,

^b^ genotype’s DSI ± SD in the field under natural epidemics,

^c^ vegetation period from planting to sample collection in days,

^d^ time period from planting to crop maturity and

^e^ species and origin (where available)

#### Culture collection and preparation of fungal inoculum

*A*. *brassicicola* isolate was collected from *Brassicaceae* crops grown at farms of FAS&T. Infected leaf samples were sterilized with 0.5% sodium hypochlorite solution for 1–2 min, subsequently by washing with distilled water up to 2–3 times. These samples were grown on potato dextrose agar (PDA) culture media and incubated at 28 °C under 12h light/dark period for 6–10 days. Identification of fungal pathogen was confirmed by analyzing slides under microscope and comparing with other *Alternaria* species relying on morphological features including shape, structure and size of conidia following Meena et al. [17].

Inoculum was prepared using potato dextrose broth (PDB) media. Fungal colony was inoculated to PDB in Erlenmeyer flask at 24 ± 1 °C and 12h light/dark period for 10 days. The inoculum concentration was determined with a hemocytometer and adjusted to 5 × 10^4^/ml following Akhtar’s [18] protocol.

### Experiments conditions

#### Bioassay optimization

Experiments were carried out under field conditions. Bioassays included photosynthetic pigments, total soluble proteins and redox enzymatic activities at different time intervals. Four *Brassicaceae* genotypes selected from screening of 150 genotypes (data shown in S1 Table) towards *A*. *brassicicola* in epidemic conditions. These consist of two *B*. *rapa* (EC250407, EC1333) and two *B*. *napus* (EC1494, EC24181) genotypes, each showing a varying degree of response to dark leaf spot were used in current study.

The 3^rd^ or 4^th^ fully expanded leaves from 40-day old plants of each genotype were inoculated with *A*. *brassicicola* and covered with polythene bags for 4–5 days to maintain high humidity (>75%). For bioassay evaluation, leaf samples were collected at different time intervals (4 hpi, 24 hpi, 48 hpi and 72 hpi).

Genotypes EC250407 and EC1494 were used as tolerant control, while EC1333 and EC24181 were susceptible controls for field mustard and rapeseed, respectively. The 45-days old symptomatic *Brassicaceae* plants were evaluated for dark leaf spot disease after inoculation. Experiment was conducted with three replicates of independent sets and each genotype was represented by three seedlings/replication or nine seedlings/genotype.

#### Field assessments

Four genotypes from *B*. *rapa* (mustard) and *B*. *napus* (rapeseed), with diverse morphological and yield contributing characteristics were evaluated under field conditions at FAS&T. Seeds were planted keeping plant-plant and row-row distances of 50 and 60 cm, respectively. The experimental design was randomized complete block with three replications. Block consisted of 10 plants in a single row. All possible cultural practices were applied until maturity. No fungicides were applied during vegetation and reproductive period for disease evaluation of genotypes.

For assessment of yield and yield linked parameters, data for plant height (cm), number of pods per plant, number of seeds per pod, raceme length (cm), silique length (cm), thousand seed weight (TSW; g) and yield per plant (g) were collected at maturity. Data for each parameter were collected from three plants per replication or nine plants per genotype.

#### Disease scoring

The degree of infection on seedlings was assessed seven days after inoculation. Field trials were evaluated regularly until plants reached maturity. Disease intensity was rated using Doullah’s model [19] with 1 to 10 scale: where 1 = no spots/chlorosis on leaf, 2 = a few pinpoint spots but no chlorosis, 3 = some spots but no large lesions/chlorosis, 4 = some spots with a few lesions enclosed by light chlorosis, 5–9 = increasing number, lesions size and chlorosis, 10 = >90% of leaf covered by lesions/chlorosis.

Consequently, genotypes with DSI of 1–4 were categorized as tolerant and those with 4.1–10 DSI were categorized as susceptible.

### Molecular and biochemical analysis

#### Leaf sampling

Thirty seedlings per each genotype were grown in the field for 40 days and inoculated with *A*. *brassicicola*. On the contrary, control leaves were inoculated with sterilized distilled water. At each time interval (4hpi, 24 hpi, 48 hpi, 72 hpi) samples were collected from nine plants per genotype or three plants per replication. Collected samples were stored at −80 °C until further analysis.

#### Estimation of photosynthesis pigments

For chlorophyll content, healthy and infected leaf samples (0.5 g) were homogenized with 80% acetone. Chlorophyll contents were estimated by measuring absorbance at 645 and 663 nm according to Arnon [20] equation for chlorophyll a, chlorophyll b, while Lichtenthaler and Wellburn [21] equation was used for total chlorophyll.

Chlorophyll“a”(mg/g)=((0.0127×A663−0.00269×A645)×100)/0.5

Chlorophyll“b”(mg/g)=((0.0229×A645−0.00468×A663)×100)/0.5

Totalchlorophyll(mg/g)=(20.2(A645)−8.02(A663))/0.5

Carotenoids were estimated by measuring absorbance at 480, 645 and 663 nm. Carotenoid amount was calculated using Davies method [22].

Carotenoid(mg/g)=(A480nm+0.114×A663nm−0.638×A645nm)

#### Assessment of total soluble proteins (TSP)

Plant leaves (0.5g) were grinded in pre-chilled pestle mortar using 1ml extraction buffer (pH 7.2). Cocktail protease inhibitor (1μM) was added to extraction buffer. Extraction phosphate buffer consists of 2.7 mM KCl, 10mM K_2_HPO_4_, 1.37mM NaCl and 2mM KH_2_PO_4_ dissolved in distilled H_2_O [23]. The ground material was centrifuged at 12000 rpm for 5 min. Supernatant was shifted to fresh centrifuge tube and preserved for protein analysis. Absorbance were measured at 595 nm and TSP were computed using standard curve by Bradford assay [24].

#### Estimation of redox-enzymatic activity

To determine redox enzymatic activity, leaf samples were ground in 5 ml of 50 mM phosphate buffer (pH 7.8). Ground material was centrifuged at 12000 rpm for 20 min. Supernatants were further used for determination of POD, CAT and SOD activity.

Guaiacol oxidation was used for the measurement of POD activity and defined as 0.01 change in absorbance/minute/mg protein. Reaction mixture was prepared by adding 2 ml phosphate buffer (50 mM), 400 μl guaiacol (20 mM), 500 μl H_2_O_2_ (40 mM) and 100 μl enzyme extract. Change in absorbance of reaction mixture was observed at 470 nm after every 20 sec for two minutes. The POD activity was expressed as IU min^-1^mg^-1^ protein [25].

The CAT activity was assayed by H_2_O_2_ decomposition. Change in absorbance due to H_2_O_2_ was observed for 2 min at 240 nm after every 20 seconds using spectrophotometer. Reaction mixture consisted of 2 ml phosphate buffer (50 mM), 900 μl H_2_O_2_ (5.9 mM) and 0.1 ml enzyme extract. The CAT activity was expressed as IU min^-1^mg^-1^ protein [25].

Photochemical reduction of nitro blue tetrazolium (NBT) was repressed by SOD activity. Inhibition by NBT was utilized to assess SOD activity. Reaction mixture was prepared by adding 950 μl phosphate buffer (50 mM), 500 μl methionine (13 mM), 500 μl EDTA (75mM), 1 ml NBT (50 μM) and 1 ml riboflavin (1.3 μM) to 50 μl enzyme extract. Reaction was initiated by keeping reaction mixture under fluorescent lamp for 5 min. Blue formazane was produced as a result of NBT photo reduction, which was used to calculate the change in absorbance at 560 nm. Same reaction mixture except enzyme extract in dark was used as blank. The SOD activity was expressed as SOD IU min^-1^ mg^-1^ protein [26].

### Biometrical analysis

Computation and data comparisons were performed using MS Excel 2016 and statistix (v.8.1) software. Descriptive statistics was employed for the calculation of means, standard deviations, medians, quartiles etc. Data comparisons were performed by two-way ANOVA (confidence level 0.95), with post-hoc Tukey’s Honestly Significant Difference (HSD; α = 0.05) analyses. The P-values for all ANOVA analyses are reported along with the respective data. To determine interaction of bioassays and morpho-yield parameters, loading plot with principal components (PC) was executed using SPSS (v.20).

## Results

**Fig 1:**
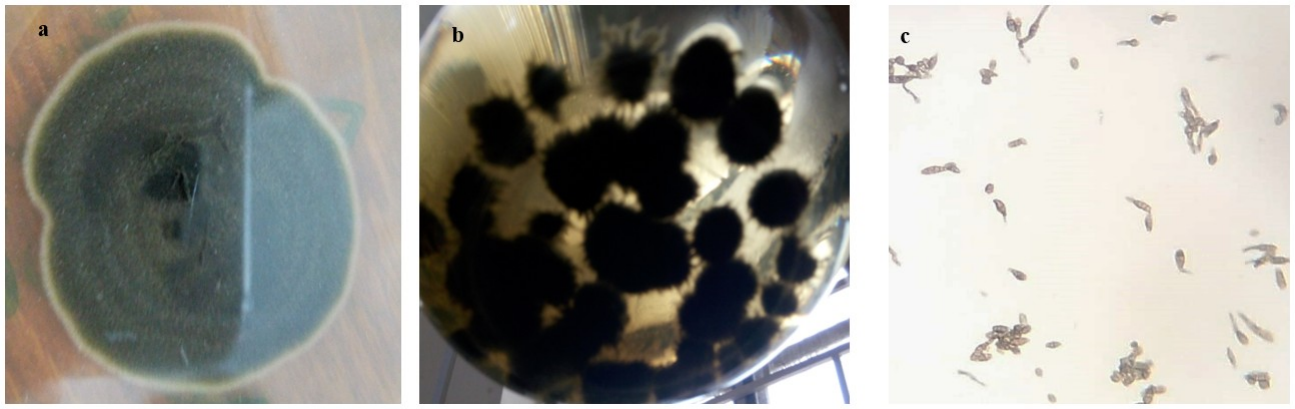
Isolation, purification and identification of *A*. *brassicicola* from *Brassicaceae*. **(a-b)** growth of *A*. *brassicicola* on PDA media (5 days) (**b)** growth of *A*. *brassicicola* on PDB media (10 days) **(c)** conidia of *A*. *brassicicola*.

Identification of *A*. *brassicicola* was performed based on microscopic analysis for the morphological features of the pathogen (Fig 1).

### Infection of *Brassica* leaves by spore suspension of *A*. *brassicicola*

**Fig 2:**
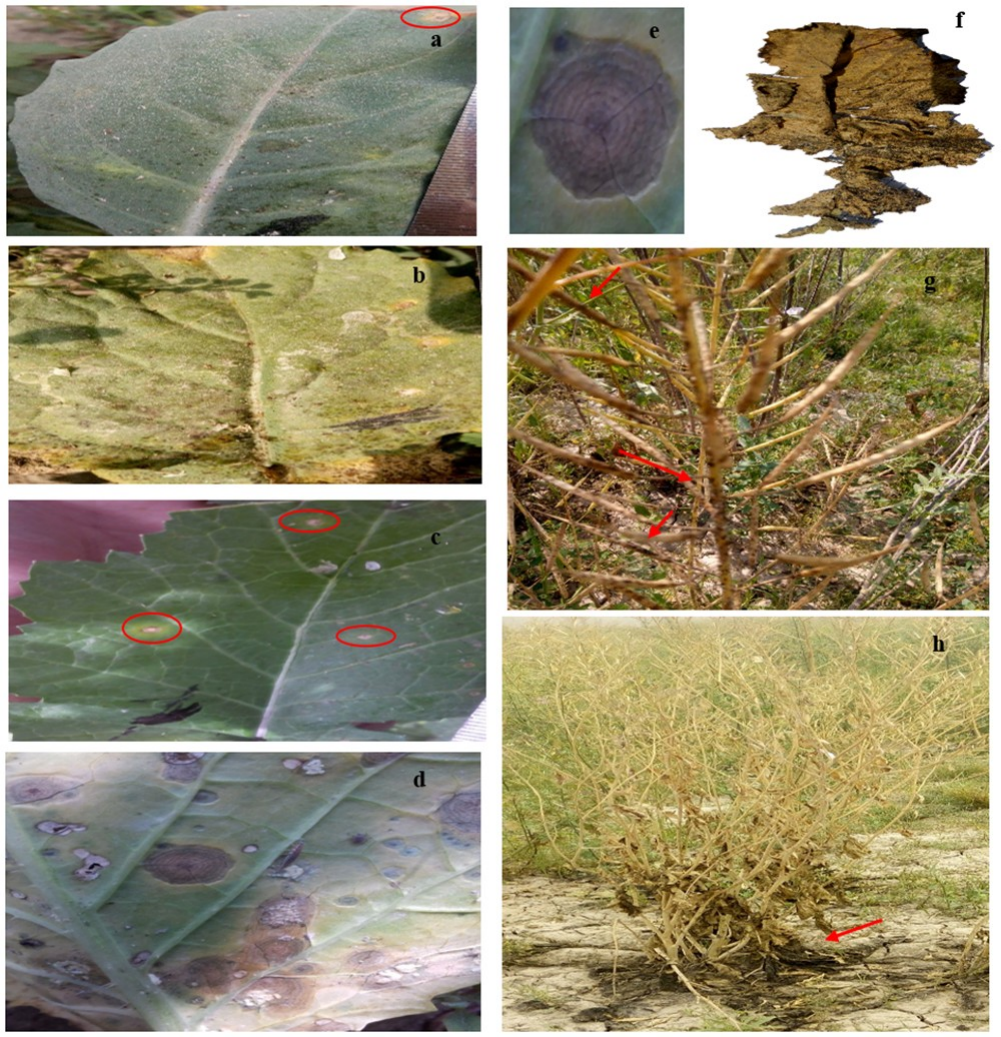
Infection on *Brassica* plants caused by *A*. *brassicicola*. **(a)** EC1494 (tolerant *B*. *napus*), **(b)** EC24181 (susceptible *B*. *napus*), **(c)** EC250407 (tolerant *B*. *rapa*), **(d)** EC1333 (susceptible *B*. *rapa*) **(e)** concentric rings surrounded by necrotic lesions **(f)** matured *Brassica* leaf covered by black sooty mold of *A*. *brassicicola* **(g)** black spots covered plant siliques and stem **(h)** plant roots and soil badly affected by black mold *of A*. *brassicicola*.

Pathological symptoms were observed in all genotypes used in the current study on exposure to the spore suspension of *A*. *brassicicola*. Although, most prominent symptoms were observed in highly susceptible genotype EC1333 (Fig 2), the highest DSI was observed in EC1333 (9.5) followed by EC24181 (5) and least in genotypes EC250407 and EC1494 (2.9 and 2.1). Spots with larger sizes were observed in susceptible genotype as compared to tolerant ones. Concentric rings surrounded by necrotic lesions were quite prominent in EC24181 (Fig 2a–2e). Genotypes EC250407 and EC1333 served as tolerant and susceptible control *for B*. *rapa*, whereas EC1494 and EC24181 served as tolerant and susceptible control, respectively for *B*. *napus* due to their varying DSI (Table 1).

### Effect on photosynthetic pigments

Chlorophyll contents decreased along with time after challenging with spore suspension of pathogen. Pathogen-treated plants lost their chlorophyll contents and became yellow. Minimum chlorophyll (chl *a*, chl *b* and total chlorophyll) contents were observed in infected leaves as compared to control. Maximum chlorophyll “*a*” reduction was observed in pathogen treated samples of EC1333 (60.5%) followed by EC24181 (58.7%), EC250407 (36.8%) and EC1494 (33.8%) at 72 hpi compared to control. Likewise, reduction in chlorophyll “*b”* was observed in leaves of EC1333 (56.9%) infected with pathogen followed by EC24181 (50.5%), EC250407 (48.3%) and EC1494 (39.8%) at 72 hpi. Total chlorophyll content also followed the same pattern of reduction, i.e., EC1333 (59.6%), EC24181 (56.6%), EC250407 (40.0%) and EC1494 (35.3%) at 72 hpi. Similar results were observed for carotenoids’ reduction, i.e., EC1333 (58.9%), EC24181 (47.5%), EC250407 (38.3%) and EC1494 (30.3%) at 72 hpi. Necrotic lesions were more prominent in susceptible genotype with minimum chlorophyll contents (Fig 2d).

### Molecular and biochemical analysis

**Fig 3:**
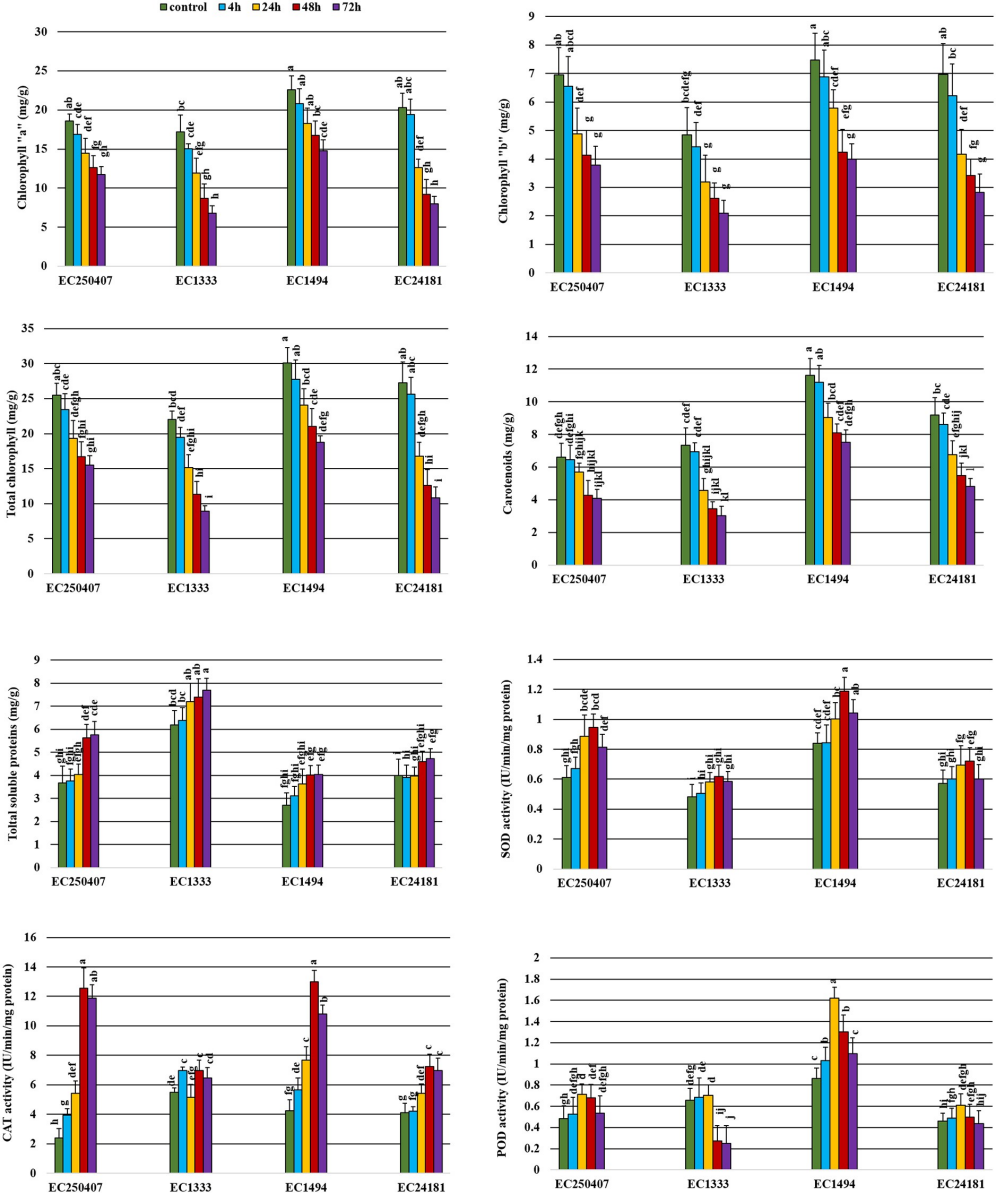
Molecular and biochemical assays of the *A*. *brassicicola*-*Brassicaceae* pathosystem. Genotypes (40-day old seedlings) were inoculated with spore suspenion. Samples were taken from 4 hpi to 72 hpi. Processed samples were used for several bioassays comprising of photosynthetic pigments, three redox enzymatic activities and total soluble proteins, with atleast three replication per sample. Bars represent means with standard deviation. Datasets for each parameter were analyzed with 2-way ANOVA (α = 0.05; S2 Table), with posthoc Tukey’s HSD whenever significant (P < 0.05) interaction between genotypes and time (hpi) were recorded. Data points with same letters do not differ significantly.

TSP and three redox enzymatic (SOD, CAT and POD) activities showed significant interaction between genotypes and post infection time (P < 0.05; Fig 3). Contrary to photosynthetic pigments, TSP contents increased with disease progression. It was observed that maximum TSP were observed in highly susceptible genotype EC1333 (7.7 mg/g) followed by EC250407 (5.8 mg/g), EC24181 (4.72 mg/g) and EC1494 (4.04 mg/g) at 72 hpi. The gradual increase in TSP was observed from 4 hpi-72 hpi (Fig 3).

Plants have developed a biochemical defense mechanism to protect themselves from phytopathogens. *A*. *brassicicola* post-infection was associated with an increase of SOD and POD activities in infected leaves. A gradual increase in SOD and POD activities with disease progression was observed in all genotypes but was more remarkable in EC250407 and EC1494 (tolerant) genotypes. Likewise, CAT showed same pattern of activity in tolerant genotypes but deviation from gradual increase was observed in susceptible genotypes i.e. EC24181 and EC1333.

### Field evaluations for morpho-yield traits

**Fig 4:**
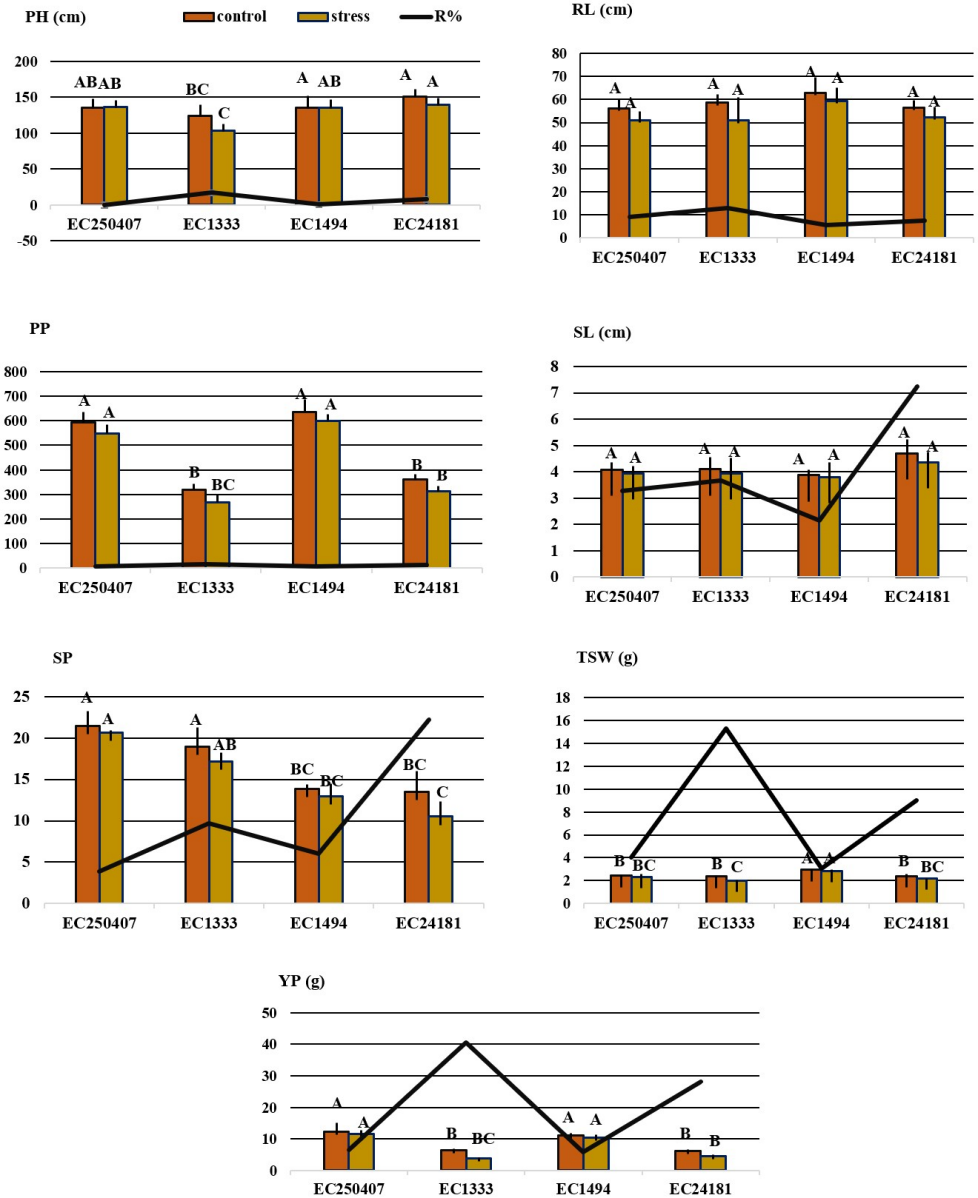
Morpho-yield parameters in rapeseed and field mustard. **Bars represent means with standard deviation**. Datasets for each parameter were analyzed with 2-way ANOVA (α = 0.05; S3 Table), with posthoc Tukey’s HSD whenever significant (P < 0.05). Data points with same letters do not differ significantly.

Symptoms of dark leaf spot on leaves, stem, siliques and whole plant were obvious (Fig 2). Data for plant height, raceme length, silique length, pods/plant, seeds/pod, TSW and yield/plant in rapeseed and mustard genotypes under stress (infected with *A*. *brassicicola*) and control (non-infected) conditions with their respective reduction percentage under stress is shown in Fig 4.

Dark leaf spot disease caused yield reduction of up to 28 and 40% in susceptible genotypes of rapeseed and mustard, respectively. Similar reduction trend in susceptible genotypes was observed for TSW and pods per plant. Among *B*. *rapa* genotypes, EC1333 showed reduction, up to 15.35% and 16.15% while among *B*. *napus*, EC24181 showed a reduction of about 9.03% and 12.88% in TSW and pods per plant, respectively. However, no significant change was observed for plant height, silique length and raceme length in infected plants of tolerant rapeseed and mustard genotypes. However, maximum reduction was observed in susceptible genotypes of rapeseed and field mustard for silique length (7.25%) and for raceme length (13.1%), respectively in contrast to each other. Likewise, maximum reduction in plant height was observed by EC1333 (*B*. *rapa*; 16.7%) in comparison to *B*. *napus*, i.e., EC24181 (7.41%). Although, seeds per pod showed no significant change in all genotypes of rapeseed and mustard except susceptible genotype of *B*. *napus* (EC24181) which showed reduction of up to 22.2%.

### Association of morpho-yield parameters with molecular and biochemical bio-assays

**Fig 5:**
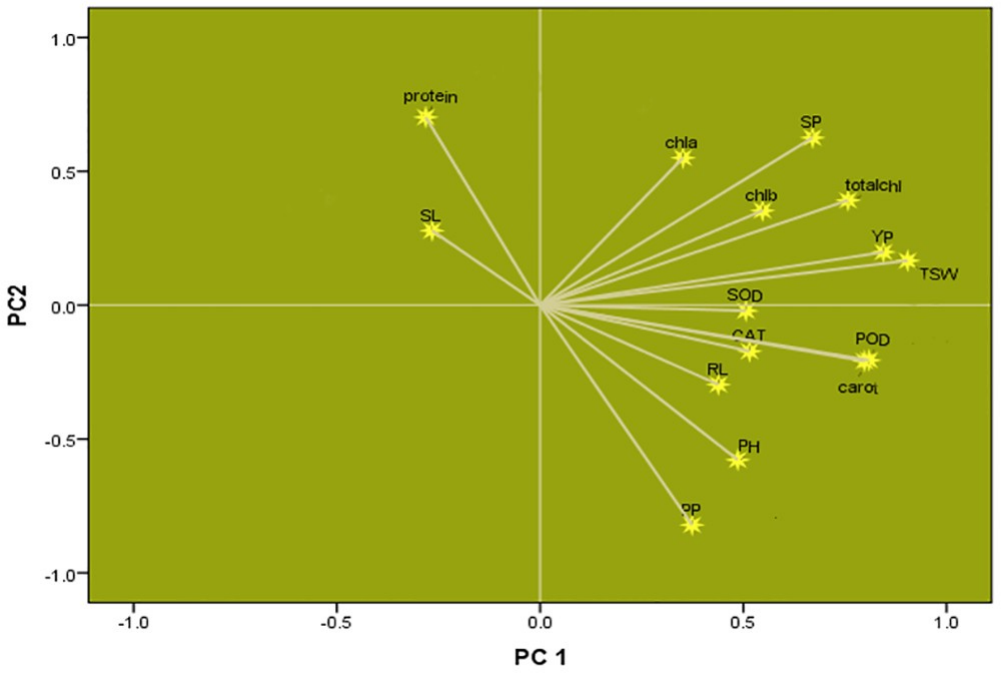
Representation of association among all studied parameters. YP: yield per plant, TSW: thousand seed weight, SL: silique length, RL: raceme length, PP: Pods per plant, PH: plant height, SP: seeds/pod, CAT: catalase, SOD: superoxide dismutase, POD: peroxidase, Carot: carotenoids, Chla: chlorophyll *a*, Chlb: Chlorophyll *b*, Totalchl: total chlorophyll, protein: Total soluble proteins.

Yield/plant showed close association with TSW, plant height, raceme length, seeds per pod, pods per plant, photosynthetic pigments and redox enzyme activities; however, negative association was observed with silique length and TSP (Fig 5).

## Discussion

Biotic stresses adversely affect plant growth and causes unfavorable variations at the cellular and molecular levels [27]. Various challenges have been encountered to discover the sources of high level tolerance against *A*. *brassicicola*, but until now no significant material is discovered [8]. However, high level tolerance has been reported in wild species of *Brassica* (reviewed by Kumar et al. [7]) but wild *Brassica* plants do not outbreed well with the cultivated susceptible ones [10]. Therefore, to find tolerant material in cultivated species, reliable tools for precise assessment of cultivars for pathogen tolerance are extremely important for any successful breeding program. To check the plant response towards disease tolerance, it is better to access germplasm under natural epidemics. Therefore, an attempt was made to test *Brassica* seedlings under natural conditions for *A*. *brassicicola* with artificial inoculum at different time intervals (4hpi, 24hpi, 48hpi, 72hpi). These results were compared with field data (un-inoculated plants/control). Inoculum concentration (5 × 10^4^/ml) as well as seedling test were quite effective for evaluation of *Brassica* genotypes towards *A*. *brassicicola* tolerance [10,28,29].

*Alternaria* fungi are known to cause damage to photosynthetic machinery of plants [30]. Chlorophyll and carotenoids pigments are necessary for plant photosynthesis, which are present in photosystems I and II and impart their role in light harvesting. Carotenoid inhibits oxidative stress by quenching singlet oxygen (^1^O_2_) and triplet chlorophyll (^3^Chl), thus protects photosynthetic machinery [31]. *Alternaria* negatively affects photosynthetic activities via necrosis development in leaves ultimately causing reduction in chlorophyll and carotenoids content [32]. Reduction in chlorophyll content on pathogen invasion indicates cell damage in canola tissues [33]. In the present investigation, photosynthetic pigments decreased with time after inoculation. It was observed that pathogen treated plants had lost their photosynthetic pigments. Minimum chlorophyll (chl ‘*a*’, chl ‘*b*’ and total chlorophyll) and carotenoid contents were observed in infected leaves of susceptible genotypes showing that tolerant genotypes have potential to retain their photosynthetic pigment under stress. These findings were supported by Martinez [34]. Borah et al. [35] proposed that reduction in chlorophyll contents (chlorophyll ‘*a*’ and ‘*b*’) might be attributed to inhibition of synthesis instead of degradation of existing pigments. Such changes in photosynthetic attributes are general signs of stress. Therefore, sustaining chlorophyll content in plants upon pathogen invasion is vital, as it will permit plant cell to continue photosynthesis.

Our result showed that TSP contents were increased with disease progression in mustard and rapeseed plants. On contrary to photosynthetic pigments, TSP contents increased in susceptible genotypes as compared to tolerant ones. Parallel trend was also observed by Onifade and Agboola [36], who suggested that proliferation of pathogen synthesize numerous enzymatic proteins and cause, occasionally, rearrangement of nutritive composition of substrate due to formation of many degraded byproducts thereby enhancing its protein content. Amino acids act as a substrate for causal pathogen during host-pathogen interplay [37]. They might also be involved in metabolic phenomenon interlinked with disease tolerance and exerting fungistatic effects [38,39] via synthesis of infection specific proteins e.g. glyceollin accumulation in soybean tissues upon pathogen invasion [40].

The oxidative burst or overproduction of ROS belongs to the earliest defense responses against pathogen invasion in plants [30]. To counter-balance the effect of oxidative stress, plants have developed an arsenal of defense mechanisms against pathogen outbreak [41]. In our case, high SOD activity was observed in all genotypes due to high ROS accumulation as SOD acts as first line of defense against oxidative burst and dismutate O_2_^-^ to H_2_O_2_ and O_2_. In current study, SOD activity was higher in tolerant genotypes. Ehsani-Moghaddam et al. [42] also observed high SOD activity in resistant strawberry genotypes against *Mycosphaerella fragariae* and concluded that resistant genotype possess higher SOD activity and contribute in efficient antioxidant mechanism. Higher SOD activity can be a selection tool for plant tolerance against diseases. Likewise, CAT and POD counter over production of H_2_O_2_ [43] and play central role in plant defense response [44]. Several evidences support defensive role of POD activity in disease tolerance mechanism against *Alternaria* [45,46] via production of phenolics, phytoalexins and glycoproteins [47]. Increase in CAT activity was observed from 4 hpi to 48 hpi and decreased after 48 hpi as shown in Fig 3. The increase in CAT activity during 0 hpi to 48 hpi showed scavenging of excessive H_2_O_2_ produced in plants [16]. Reduction in CAT activity occur after 48 hpi due to over production/accretion of H_2_O_2_ and might be due to enhanced proteolysis induced by oxidative burst [48]. Durner and Klessig [49] reported that decrease in CAT activity was a part of plant defense response to protect it against pathogens and plant can tolerate excessive H_2_O_2_ concentration when CAT activity was least. Although, our results showed that CAT activity was higher and more obvious in tolerant genotypes as compared to susceptible genotypes depicting the role of CAT in tolerance mechanism; as also reported by Meena et al. [17] and Debona et al. [50].

Yield losses of 28–40% by dark leaf spot were observed in current investigation, which is a major cause of yield reduction in oilseed *Brassica*. Thus, yield improvement depends on extent of genetic variability for different traits as well as on disease tolerance. Hence, estimation of magnitude of genetic variability for yield parameters is obligatory for overall yield improvement [51].

Tracking the association between different traits by presenting various features of plant growth and regulation could provide clear perceptions about different interactions among plants and help researchers to use this data for better management practices and to make development in plant production [52]. To prompt diverse aspect of association of several yield variables, different biometrical techniques have been developed. The PC analysis helps researchers to use simple illustrations such as factors loading to describe relationships among each variable under study [52,53]. In the present study, factor loading graph showed close association of yield with different morpho-biochemical and photosynthetic pigments. Redox-enzymatic antioxidants showed positive association among themselves as well as with plant yield which depict their role in plant yield improvement by means of disease tolerance. Higher enzyme activities are involved in disease tolerance and ultimately to better yield under *Alternaria* stress. Poli et al. [54] also observed significant association between redox enzymes (POD, CAT, SOD) and plant yield.

Commercial seed yield parameters were explored to determine the final seed yield. From the loading plot, it became clear that parameters previously used as a substitution for yield/plant, such as the seeds/pod, plant height, pods per plant, TSW and raceme length [55, 56] were significantly associated with yield/plant in this study (Fig 5) but not with silique length. These results suggest that commercially-used proxy, such as silique length, may not be the significant gauge of yield for breeding and selection purposes [56,57]. Silique length had negative relationship with other morpho-biochemical; yield linked parameters except TSP. As discussed earlier, TSP increases with disease progression; contrary to yield thus having negative association with yield/defense mechanism (redox enzymes) but association of silique length with yield is still unresolved. Although, dark leaf spot did not significantly reduce plant height, pods/plant, silique length or raceme length but infected *Brassica* pods failed to develop healthy seeds. Thus, severely spotted pods became dry, shrunk, and shattered pre-maturely, letting shrunken seeds to drop off [58]. However, yield was positively associated with seeds/pod and TSW. There is a need to invest in a better plant survial strategy to develop pods with healtheir or more seeds rather than investing time on *Brassica* breeding for silique length; a similar view has been reported earlier in rapeseed [59].

*Brassica* yield had positive association with chlorophyll ‘*a*’, chlorophyll ‘*b*’, total chlorophyll and carotenoids supporting the statement that “plant growth/yield and photosynthetic pigments are interlinked” as was proposed previously [60]. Thus, any constraint in photosynthesis disturbs plant growth and ultimately leads to yield reduction [61,62].

## Conclusion

Genetic control of rapeseed and mustard genotypes tolerant against *A*. *brassicicola* plays a critical role in determining the patho-system interaction outcome. Our study on assessment of *A*. *brassicicola* tolerance among rapeseed and mustard revealed that dark leaf spot disease induced an extreme alteration in plant biochemistry that cause reduction in photosynthetic area, defoliation, accelerated senescence and ultimately poor yield in susceptible genotypes as compared to tolerant ones. Physio-biochemical defense response, as evidenced by tolerant genotypes via augmented activities of defense enzymes, is a vital sign of their role in *Brassica*-*Alternaria* interactions and its tolerance. Currently, these physiological, morpho-biochemical marker indices can be used as probes for rapid screening of germplasm. Although, moderate tolerant germplasm EC250407 (mustard) and EC1494 (rapeseed) can be utilized for future experiments and can serves as tolerant material for black spot disease.

## Supporting information

S1 Table Screening of Brassica genotypes against dark leaf spot disease with their accession numbers and disease severity index. (DOCX)

S2 Table Analysis of variance of bio-assay in rapeseed and field mustard against dark leaf spot disease. **: Significant at 1% probability level; *: Significant at 5% probability level; chl a: chlorophyll a; chl b: chlorophyll b; total chl: total chlorophyll; carot: carotenoids; TSP: total soluble proteins; SOD: superoxide dismutase; POD: peroxidase; CAT: catalase.
(DOCX)

S3 Table Analysis of variance of morpho-yield parameters in rapeseed and field mustard against dark leaf spot disease. **: Significant at 1% probability level; *: Significant at 5% probability level; YP: yield per plant, TSW: thousand seed weight, SL: silique length, RL: raceme length, PP: Pods per plant, PH: plant height, SP: seeds/pod.
(DOCX)
