# Supplementary material for: Acuities into tolerance mechanisms via different bioassay during Brassicaceae-Alternaria brassicicola interaction and its impact on yield
Source: PLoS One. 2020 Dec 1;15(12):e0242545. doi: 10.1371/journal.pone.0242545 (PMC7707606; doi:10.1371/journal.pone.0242545)
Supplement: S1 Table — (DOCX) [file pone.0242545.s001.docx]

**S1 Table: Screening of *Brassica* genotypes against dark leaf spot disease with their accession numbers and disease severity index**

| Genotype | | | | DSI ± SD | | | Genetic background | | | | Genotype | | | | DSI ± SD | | | | | | Genetic background | | | |
| --- | --- | --- | --- | --- | --- | --- | --- | --- | --- | --- | --- | --- | --- | --- | --- | --- | --- | --- | --- | --- | --- | --- | --- | --- |
| EC928 | | | | 3.6 ± 0.19 | | | *B. rapa* | | | | **EC1483** | | | | 3.3 ± 0.33 | | | | | | | *B. rapa* | | |
| EC25047 | | | | 2.9 ± 0.19 | | | *B. rapa* | | | | **EC1484** | | | | 3.2 ± 0.33 | | | | | | | *B. rapa* | | |
| EC1333 | | | | 9.35 ± 0.39 | | | *B. rapa* | | | | **EC1485** | | | | 5.5 ± 0.19 | | | | | | | *B. rapa* | | |
| EC1341 | | | | 4.6 ± 0.38 | | | *B. rapa* | | | | **EC1490** | | | | 5.3 ± 0.33 | | | | | | | *B. rapa* | | |
| EC1347 | | | | 7.0 ± 0.33 | | | *B. rapa* | | | | **EC1497** | | | | 5.5 ± 0.19 | | | | | | | *B. rapa* | | |
| EC1350 | | | | 4.7 ± 0.33 | | | *B. rapa* | | | | **EC1498** | | | | 3.2 ± 0.19 | | | | | | | *B. rapa* | | |
| EC1354 | | | | 4.1 ± 0.19 | | | *B. rapa* | | | | **EC1501** | | | | 3.2 ± 0.33 | | | | | | | *B. rapa* | | |
| EC1368 | | | | 6.5 ± 0.19 | | | *B. rapa* | | | | **EC1601** | | | | 8.0 ± 0.33 | | | | | | | *B. rapa* | | |
| EC1374 | | | | 4.2 ± 0.38 | | | *B. rapa* | | | | **EC1603** | | | | 5.0 ± 0.38 | | | | | | | *B. rapa* | | |
| EC1381 | | | | 6.4 ± 0.51 | | | *B. rapa* | | | | **EC1712** | | | | 8.4 ± 0.19 | | | | | | | *B. rapa* | | |
| EC1388 | | | | 2.8 ± 0.33 | | | *B. rapa* | | | | **EC1714** | | | | 7.0 ± 0.38 | | | | | | | *B. rapa* | | |
| EC1389 | | | | 3.2 ± 0.33 | | | *B. rapa* | | | | **EC25016** | | | | 3 ± 0.33 | | | | | | | *B. rapa* | | |
| EC1418 | | | | 5.4 ± 0.51 | | | *B. rapa* | | | | **EC25017** | | | | 4.0 ± 0.33 | | | | | | | *B. rapa* | | |
| EC1471 | | | | 4.0 ± 0.33 | | | *B. rapa* | | | | **EC25018** | | | | 5.9 ± 0.69 | | | | | | | *B. rapa* | | |
| EC1477 | | | | 2.4 ± 0.33 | | | *B. rapa* | | | | **EC25022** | | | | 6.4 ± 0.67 | | | | | | | *B. rapa* | | |
| EC1478 | | | | 5.3 ± 0.33 | | | *B. rapa* | | | | **EC25023** | | | | 7.1 ± 0.69 | | | | | | | *B. rapa* | | |
| EC1479 | | | | 6.7 ± 0.58 | | | *B. rapa* | | | | **EC25030** | | | | 5.6 ± 0.33 | | | | | | | *B. rapa* | | |
| EC1480 | | | | 6.3 ± 0.33 | | | *B. rapa* | | | | **EC25034** | | | | 6.8 ± 0.51 | | | | | | | *B. rapa* | | |
| EC1481 | | | | 5.0 ± 0.33 | | | *B. rapa* | | | | **EC25035** | | | | 5.7 ± 0.19 | | | | | | | *B. rapa* | | |
| EC1482 | | | | 4.3 ± 0.33 | | | *B. rapa* | | | | **EC25042** | | | | 7.1 ± 0.51 | | | | | | | *B. rapa* | | |
| Genotype | | **DSI ± SD** | | | | **Genetic background** | | | **Genotype** | | | | **DSI ± SD** | | | | | | | **Genetic background** | | | |  |
| EC25045 | | 3.4 ± 0.19 | | | | *B. rapa* | | | **EC26266** | | | | 2.7 ± 0.33 | | | | | | | *B. rapa* | | | |  |
| EC25044 | | 7.6 ± 0.51 | | | | *B. rapa* | | | **EC26268** | | | | 4.7 ± 0.58 | | | | | | | *B. rapa* | | | |  |
| EC25045 | | 6.0 ± 0.33 | | | | *B. rapa* | | | **EC26269** | | | | 6.9 ± 0.19 | | | | | | | *B. rapa* | | | |  |
| EC25048 | | 5.0 ± 0.38 | | | | *B. rapa* | | | **EC26270** | | | | 6.2 ± 0.84 | | | | | | | *B. rapa* | | | |  |
| EC25049 | | 6.0 ± 0.38 | | | | *B. rapa* | | | **EC26271** | | | | 5.9 ± 0.19 | | | | | | | *B. rapa* | | | |  |
| EC25050 | | 6.4 ± 0.51 | | | | *B. rapa* | | | **EC26274** | | | | 4.2 ± 0.33 | | | | | | | *B. rapa* | | | |  |
| EC25055 | | 3.0 ± 0.33 | | | | *B. rapa* | | | **EC1494** | | | | 2.1 ± 0.19 | | | | | | | *B. napus* | | | |  |
| EC25065 | | 5.2 ± 0.38 | | | | *B. rapa* | | | **EC1500** | | | | 3.2 ± 0.19 | | | | | | | *B. napus* | | | |  |
| EC25081 | | 5.1 ± 0.38 | | | | *B. rapa* | | | **EC1692** | | | | 3.0 ± 0.33 | | | | | | | *B. napus* | | | |  |
| EC26156 | | 4.8 ± 0.19 | | | | *B. rapa* | | | **EC1716** | | | | 3.0 ± 0.19 | | | | | | | *B. napus* | | | |  |
| EC26157 | | 3.6 ± 0.38 | | | | *B. rapa* | | | **EC24171** | | | | 5.6 ± 0.38 | | | | | | | *B. napus* | | | |  |
| EC26158 | | 5.3 ± 0.33 | | | | *B. rapa* | | | **EC24174** | | | | 6.6 ± 0.19 | | | | | | | *B. napus* | | | |  |
| EC26159 | | 4.3 ± 0.33 | | | | *B. rapa* | | | **EC24175** | | | | 6.0 ± 0.33 | | | | | | | *B. napus* | | | |  |
| EC26161 | | 4.7 ± 0.51 | | | | *B. rapa* | | | **EC24176** | | | | 4.0 ± 0.33 | | | | | | | *B. napus* | | | |  |
| EC26166 | | 3.7 ± 0.33 | | | | *B. rapa* | | | **EC24177** | | | | 4.9 ± 0.51 | | | | | | | *B. napus* | | | |  |
| EC26171 | | 6.9 ± 0.19 | | | | *B. rapa* | | | **EC24178** | | | | 3.7 ± 0.33 | | | | | | | *B. napus* | | | |  |
| EC26172 | | 4.7 ± 0.33 | | | | *B. rapa* | | | **EC24179** | | | | 6.2 ± 0.38 | | | | | | | *B. napus* | | | |  |
| EC26173 | | 8.1 ± 0.51 | | | | *B. rapa* | | | **EC24180** | | | | 3.2 ± 0.51 | | | | | | | *B. napus* | | | |  |
| EC26174 | | 6.6 ± 0.19 | | | | *B. rapa* | | | **EC24181** | | | | 5.0 ± 0.33 | | | | | | | *B. napus* | | | |  |
| EC26177 | | 4.8 ± 0.88 | | | | *B. rapa* | | | **EC24182** | | | | 3.4 ± 0.69 | | | | | | | *B. napus* | | | |  |
| EC26263 | | 7.3 ± 0.00 | | | | *B. rapa* | | | **EC24183** | | | | 7.4 ± 0.69 | | | | | | | *B. napus* | | | |  |
| Genotype | | | **DI ± SD** | | | **Genetic background** | | **Genotype** | | | | **DI ± SD** | | | | **Genetic background** | | | | | | | |  |
| EC24184 | | | 5.7 ± 0.58 | | | *B. napus* | | **EC1495** | | | | 4.4 ± 0.33 | | | | *B. juncea* | | | | | | | |  |
| EC24185 | | | 7.3 ± 0.33 | | | *B. napus* | | **EC1496** | | | | 5.6 ± 0.58 | | | | *B. juncea* | | | | | | | |  |
| EC24186 | | | 2.6 ± 0.19 | | | *B. napus* | | **EC1662** | | | | 3.1 ± 0.69 | | | | *B. juncea* | | | | | | | |  |
| EC24187 | | | 4.5 ± 0.33 | | | *B. napus* | | **EC1703** | | | | 4.6 ± 0.33 | | | | *B. juncea* | | | | | | | |  |
| EC24189 | | | 7.6 ± 0.38 | | | *B. napus* | | **EC1709** | | | | 2.8 ± 0.19 | | | | *B. juncea* | | | | | | | |  |
| EC24190 | | | 6.7 ± 0.33 | | | *B. napus* | | **EC1710** | | | | 3.5 ± 0.19 | | | | *B. juncea* | | | | | | | |  |
| EC24191 | | | 3.8 ± 0.38 | | | *B. napus* | | **EC1713** | | | | 4.7 ± 0.69 | | | | *B. juncea* | | | | | | | |  |
| EC24192 | | | 3.4 ± 0.38 | | | *B. napus* | | **EC19507** | | | | 5.8 ± 0.38 | | | | *B. juncea* | | | | | | | |  |
| EC24193 | | | 4.7 ± 0.19 | | | *B. napus* | | **EC19520** | | | | 4.4 ± 0.33 | | | | *B. juncea* | | | | | | | |  |
| EC24194 | | | 2.3 ± 0.33 | | | *B. napus* | | **EC24047** | | | | 4.6 ± 0.19 | | | | *B. juncea* | | | | | | | |  |
| EC24195 | | | 6.0 ± 0.33 | | | *B. napus* | | **EC24048** | | | | 3.5 ± 0.33 | | | | *B. juncea* | | | | | | | |  |
| EC24196 | | | 3.8 ± 0.38 | | | *B. napus* | | **EC24049** | | | | 2.3 ± 0.33 | | | | *B. juncea* | | | | | | | |  |
| EC1060 | | | 4.8 ± 0.33 | | | *B. juncea* | | **EC24050** | | | | 3.2 ± 0.33 | | | | *B. juncea* | | | | | | | |  |
| EC1327 | | | 2.4 ± 0.19 | | | *B. juncea* | | **EC24051** | | | | 5.6 ± 0.19 | | | | *B. juncea* | | | | | | | |  |
| EC1342 | | | 5.0 ± 0.33 | | | *B. juncea* | | **EC24052** | | | | 2.1 ± 0.19 | | | | *B. juncea* | | | | | | | |  |
| EC1358 | | | 4.6 ± 0.33 | | | *B. juncea* | | **EC24053** | | | | 3.2 ± 0.33 | | | | *B. juncea* | | | | | | | |  |
| EC1359 | | | 6.0 ± 0.19 | | | *B. juncea* | | **EC24054** | | | | 2.6 ± 0.38 | | | | *B. juncea* | | | | | | | |  |
| EC1412 | | | 2.5 ± 0.19 | | | *B. juncea* | | **EC24055** | | | | 3.1 ± 0.19 | | | | *B. juncea* | | | | | | | |  |
| Genotype | **DI ± SD** | | | | **Genetic background** | | | | | **Genotype** | | | | **DI ± SD** | | | | | **Genetic background** | | | | |  |
| EC24056 | 5.6 ± 0.19 | | | | *B. juncea* | | | | | **EC25925** | | | | 2.8 ± 0.51 | | | | *B. carinata* | | | | | |  |
| EC24057 | 8.0 ± 0.88 | | | | *B. juncea* | | | | | **EC25926** | | | | 2.0 ± 0.33 | | | | *B. carinata* | | | | | |  |
| EC24058 | 3.2 ± 0.33 | | | | *B. juncea* | | | | | **EC25928** | | | | 4.4 ± 0.19 | | | | *B. carinata* | | | | | |  |
| EC24059 | 5.7 ± 0.00 | | | | *B. juncea* | | | | | **EC25929** | | | | 2.7 ± 0.33 | | | | *B. carinata* | | | | | |  |
| EC24060 | 3.8 ± 0.19 | | | | *B. juncea* | | | | | **EC25930** | | | | 3.6 ± 0.19 | | | | *B. carinata* | | | | | |  |
| EC24061 | 2.6 ± 0.51 | | | | *B. juncea* | | | | | **EC25931** | | | | 3.4 ± 0.19 | | | | *B. carinata* | | | | | |  |
| EC24062 | 6.0 ± 0.58 | | | | *B. juncea* | | | | | **EC25933** | | | | 2.4 ± 0.19 | | | | *B. carinata* | | | | | |  |
| EC24063 | 2.9 ± 0.19 | | | | *B. juncea* | | | | | **EC25935** | | | | 2.6 ± 0.19 | | | | *B. carinata* | | | | | |  |
| EC24065 | 5.3 ± 0.33 | | | | *B. juncea* | | | | | **EC25936** | | | | 2.4 ± 0.19 | | | | *B. carinata* | | | | | |  |
| EC24066 | 6.3 ± 0.33 | | | | *B. juncea* | | | | | **EC25937** | | | | 2.8 ± 0.19 | | | | *B. carinata* | | | | | |  |
| EC24067 | 2.0 ± 0.33 | | | | *B. juncea* | | | | | **EC25938** | | | | 4.3 ± 0.19 | | | | *B. carinata* | | | | | |  |
| EC24068 | 8.4 ± 0.51 | | | | *B. juncea* | | | | | **EC25939** | | | | 6.3 ± 0.58 | | | | *B. carinata* | | | | | |  |
| EC24069 | 8.8 ± 0.19 | | | | *B. juncea* | | | | | **EC25940** | | | | 4.3 ± 0.67 | | | | *B. carinata* | | | | | |  |
| EC24070 | 4.4 ± 0.38 | | | | *B. juncea* | | | | | **EC25941** | | | | 3.0 ± 0.33 | | | | *B. carinata* | | | | | |  |
| EC24071 | 6.4 ± 0.19 | | | | *B. juncea* | | | | | **EC25943** | | | | 2.0 ± 0.33 | | | *B. carinata* | | | | | |  |  |
| EC24072 | 7.8 ± 0.69 | | | | *B. juncea* | | | | | **EC25944** | | | | 2.7 ± 0.33 | | | *B. carinata* | | | | | |  |  |
