# Supplementary material for: Acuities into tolerance mechanisms via different bioassay during Brassicaceae-Alternaria brassicicola interaction and its impact on yield
Source: PLoS One. 2020 Dec 1;15(12):e0242545. doi: 10.1371/journal.pone.0242545 (PMC7707606; doi:10.1371/journal.pone.0242545)
Supplement: S2 Table — **: Significant at 1% probability level; *: Significant at 5% probability level; chl a: chlorophyll a; chl b: chlorophyll b; total chl: total chlorophyll; carot: carotenoids; TSP: total soluble proteins; SOD: superoxide dismutase; POD: peroxidase; CAT: catalase. (DOCX) [file pone.0242545.s002.docx]

**S2 Table: Analysis of variance of bio-assay in rapeseed and field mustard against dark leaf spot disease**

| **S.O.V** | **d_f_** | **Chl “a”** | **Chl “b”** | **Total chl** | **Carot** | **TSP** | **SOD** | **POD** | **CAT** |
| --- | --- | --- | --- | --- | --- | --- | --- | --- | --- |
| **Genotypes** | 3 | 128.67****** | 7.46****** | 183.76****** | 44.95****** | 31.35****** | 0.531****** | 2.156****** | 20.88****** |
| **Treatments** | 4 | 206.69****** | 30.92****** | 396.20****** | 44.86** | 4.04****** | 0.119****** | 0.185****** | 76.96****** |
| **Genotypes × treatments** | 12 | 5.13***** | 0.46 | 5.55 | 1.38***** | 0.64 | 0.0094****** | 0.0546****** | 12.01****** |
| **Error** | 40 | 2.55 | 0.70 | 4.14 | 0.62 | 0.15 | 0.0031 | 0.0039 | 0.153 |
| **C.V.** |  | 11.7 | 18.85 | 10.78 | 12.18 | 8.2 | 7.56 | 8.59 | 5.73 |

**: Significant at 1% probability level; *: Significant at 5% probability level; chl a: chlorophyll a; chl b: chlorophyll b; total chl: total chlorophyll; carot: carotenoids; TSP: total soluble proteins; SOD: superoxide dismutase; POD: peroxidase; CAT: catalase
