# Supplementary material for: Acuities into tolerance mechanisms via different bioassay during Brassicaceae-Alternaria brassicicola interaction and its impact on yield
Source: PLoS One. 2020 Dec 1;15(12):e0242545. doi: 10.1371/journal.pone.0242545 (PMC7707606; doi:10.1371/journal.pone.0242545)
Supplement: S3 Table — **: Significant at 1% probability level; *: Significant at 5% probability level; YP: yield per plant, TSW: thousand seed weight, SL: silique length, RL: raceme length, PP: Pods per plant, PH: plant height, SP: seeds/pod. (DOCX) [file pone.0242545.s003.docx]

**S3 Table: Analysis of variance of morpho-yield parameters in rapeseed and field mustard against dark leaf spot disease**

| **S.O.V** | **d_f_** | **PH** | **RL** | **PP** | **SL** | **SP** | **TSW** | **YP** |
| --- | --- | --- | --- | --- | --- | --- | --- | --- |
| **Genotypes** | 3 | 1143** | 72.417 | 160472** | 0.543 | 105.538** | 0.604** | 75.84** |
| **Treatments** | 4 | 433.5* | 160.67* | 12105** | 0.189 | 15.844* | 0.219** | 12.56** |
| **Genotypes × treatments** | 12 | 136.25 | 4.861 | 56 | 0.196 | 1.594 | 0.024 | 1.2464** |
| **Error** | 40 | 85.62 | 32.612 | 1175 | 0.178 | 2.688 | 0.017 | 1.3844 |
| **C.V.** |  | 6.95 | 10.67 | 7.15 | 10.28 | 10.15 | 5.35 | 14.18 |

**: Significant at 1% probability level; *: Significant at 5% probability level; PH: plant height, RL: raceme length, PP: Pods per plant, SL: silique length, SP: seeds/pod, TSW: thousand seed weight, YP: yield per plant
